# Rethinking Social Support and Conflict: Lessons from a Study of Women Who Have Separated from Abusive Partners

**DOI:** 10.1155/2012/738905

**Published:** 2012-09-02

**Authors:** Sepali Guruge, Marilyn Ford-Gilboe, Joan Samuels-Dennis, Colleen Varcoe, Piotr Wilk, Judith Wuest

**Affiliations:** ^1^School of Nursing, Ryerson University, Toronto, ON, Canada M5B 2K3; ^2^Arthur Labatt Family School of Nursing and Department of Family Medicine, Schulich School of Medicine & Dentistry, Western University, London, ON, Canada N6A 3C1; ^3^School of Nursing, York University, Toronto, ON, Canada M3I 1P3; ^4^School of Nursing, University of British Columbia, Vancouver, BC, Canada V6T 2B5; ^5^Department of Epidemiology and Pediatrics, Children's Health Research Institute, Schulich School of Medicine & Dentistry, Western University, London, ON, Canada N6A 3C1; ^6^Faculty of Nursing, University of New Brunswick, Fredericton, NB, Canada E3B 5A3

## Abstract

Relationships have both positive and negative dimensions, yet most research in the area of intimate partner violence (IPV) has focused on social support, and not on social conflict. Based on the data from 309 English-speaking Canadian women who experienced IPV in the past 3 years and were no longer living with the abuser, we tested four hypotheses examining the relationships among severity of past IPV and women's social support, social conflict, and health. We found that the severity of past IPV exerted direct negative effects on women's health. Similarly, both social support and social conflict directly influenced women's health. Social conflict, but not social support, mediated the relationships between IPV severity and health. Finally, social conflict moderated the relationships between social support and women's health, such that the positive effects of social support were attenuated in the presence of high levels of social conflict. These findings highlight that routine assessments of social support and social conflict and the use of strategies to help women enhance support and reduce conflict in their relationships are essential aspects of nursing care.

## 1. Introduction

Intimate partner violence (IPV) is a global health and social problem, which occurs in all economic, social, religious, and cultural groups and has tremendous personal and social costs for women and society at large [1]. Defined as a pattern of physical, sexual, and/or emotional abuse by a current or former intimate partner or spouse in the context of coercive control [2], IPV has been consistently linked to a wide range of physical and mental health problems that may persist long after the abuse has ended [3–6]. A priority for emerging research is identifying the mechanisms by which IPV affects health [7]. 

Women's social relationships may partially explain the relationship between IPV and health. In the context of IPV, social support has been positively associated with women's mental and physical health [8–12] and has been found to both mediate [13] and/or moderate [14] the relationship between IPV and mental health. However, few studies have examined the relationships between social support and physical health among women who have experienced IPV. 

 Social relationships often have negative dimensions or costs, but this “darker side” of relationships has received much less attention than the supportive aspects. Qualitative studies [15–17] suggest that female survivors of IPV experience demands and expectations from those in their social networks that can erode their sense of control, making it more difficult to deal with the abuse and/or build a new life after leaving an abusive partner. However, few studies have focused on whether social conflict experienced by the women limits the positive impacts of social support on health outcomes. A clearer understanding of the roles that social support and social conflict play in the health of women who have experienced IPV is needed so that health promoting services, programs, and policies can be improved. The purpose of this analysis was to examine the relationships among severity of IPV, social support, social conflict, and mental and physical health among women who have recently separated from an abusive partner. 

## 2. Background

 Social support, the perception that one has access to informational, emotional, psychological, financial, and/or instrumental aid [18], has been associated with positive health outcomes in wide range of populations [19]. Despite more than three decades of research, the mechanisms by which social support affects health are still unclear [20]. Three possible mechanisms have been proposed [21]. Social support may: (a) affect health *directly* (i.e., such that increased social support is linked to more positive health outcomes); (b) *mediate* the relationship between stress and health (i.e., higher levels of stress erode social support, resulting in poorer health); or (c) *moderate* the relationship between stress and health (i.e., the relationship between stress and health outcomes depends on the level of social support). The extent to which these 3 mechanisms have been tested in women who have experienced IPV varies. 

 Many studies have focused on the direct impact of social support on the health of women who have experienced violence. Among female survivors of IPV, social support is associated with better general health [11], fewer symptoms of posttraumatic stress disorder (PTSD) [22, 23], and reduced physical and psychological distress [9, 10] and suicidal ideation and actions [14, 24]. Social support has also been associated with lower levels of depression in women six months after leaving a shelter [8] and among abused women whose cases had gone to court [12]. 

There is also some evidence that social support mediates the relationship between IPV and women's general mental health [25], depression, anxiety, self-esteem, and PTSD [26], as well as the relationship between changes in abuse and depression over a two-year period [13]. Glass et al. [27] found that tangible support (e.g., financial help) mediated the relationships between sexual or physical IPV and PTSD symptoms, while Coker et al. [11] found that emotional support mediated the relationship between IPV and *both* physical and mental health. 

The few studies which have examined the moderating or buffering effect of social support on the relationship between IPV and women's health have produced contradictory results. Kaslow et al. [14] found that social support buffered the relationship between IPV and suicide attempts. Carlson et al. [28] found that more protective factors (including social support) buffered the effects of lifetime abuse on mental health, but not in the expected direction: women with the most lifetime abuse were least likely to experience mental health benefits from protective factors. However, they did not examine social support as a separate protective factor. Beeble et al. [13] reported that social support moderated the relationship between psychological abuse and quality of life, but not the relationships between physical or psychological abuse and depression. 

 Tilden et al. [29] propose that “support reciprocation, burden, and conflict are ubiquitous in human relationships” (page 337). However, a limited body of research has examined how these negative aspects of social relationships affect health [20]. A distinct concept, social conflict reflects the level of tension, discord, and/or stress within one's relationships, and not merely the absence of social support [30]. Evidence of the negative association between social conflict and health first emerged in the 1980s (e.g., [31–34]). Given that social conflict is often a better predictor of health outcomes than the perceived support [35, 36], the benefits of social support need to be understood in the context of costs or conflict. Although social conflict has been proposed to moderate the positive impact of support on health [20, 31], this has not been systematically studied. 

Qualitative studies of female survivors of IPV suggest that social conflict is a common experience. Family, friends, and neighbours often help women by providing refuge and resources, but may also undermine women's decision making by minimizing the abuse, blaming the women, and/or maintaining secrecy regarding IPV [37–40]. Helpers who reduce the complexity of abusive intimate relationships to incidents of violence leave women silenced and discouraged from seeking further help [15]. Community norms and values may force women to keep their experiences of abuse secret [17, 41, 42], and family and friends may side with the abuser, criticize women's choices, and tell them what to do, or refuse to provide practical assistance [43], leading to tension or overt conflict within women's social networks. Further, offers of support from family, friends, and professionals may come with “conditions” that force women to take unwanted actions [15, 16]. These intrusive costs of relationships interfere with women's abilities to promote their health and the health of their families and to build a better life after leaving an abusive partner [44]. However, little empirical evidence supports the negative impact of social conflict on health outcomes among women who have experienced IPV. Goodkind et al. [45] found that social conflict was positively associated with symptoms of depression, but this association was no longer significant when controlling for level of tangible support. 

 In summary, there is evidence that social support exerts both direct and mediating effects on the health of women who have experienced IPV, particularly in terms of mental health, but limited evidence suggests that social support moderates the impact of IPV on health. In most studies, women were either in an abusive relationship or had recently ended the relationship, raising questions about whether the findings apply to women who have been separated from an abusive partner for longer periods of time. Additionally, despite qualitative evidence that women who have been in abusive relationships may experience significant levels of social conflict, neither its direct impact on health nor its mediating effect on the relationship between IPV and health has been studied. 

### 2.1. Conceptual Model

**Figure 1:**
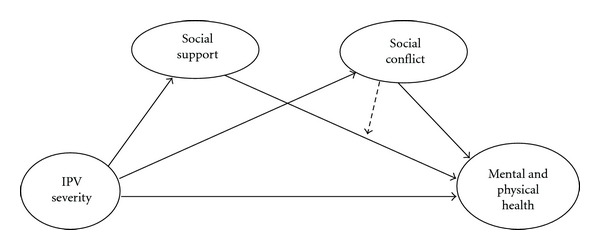
Conceptual model of hypothesized relationships among IPV severity, social support, social conflict and women's health.

Based on previous literature, we developed a conceptual model specifying the direct, mediating, and moderating effects of social support and conflict in the context of IPV and health (Figure 1). Direct and mediating effects are depicted as solid lines and the moderating effect of social conflict on the relationship between social support and health is shown as a broken line. Based on this model, we tested four hypotheses. Hypotheses 1 and 2 were that social support and conflict, each mediates the relationship between IPV severity and health. Hypothesis 3 was that social support and social conflict, each mediates the relationship between IPV and health, after controlling for the effect of the other variable. Hypothesis 4 was that social conflict moderates the relationship between social support and health, so the impact of social support on health depends on the level of social conflict present (the positive impact of social support would be weaker in the presence of high levels of conflict and vice versa). Although we acknowledge that mental and physical health are dimension of a more general health construct, understanding whether social support and social conflict function in similar or different ways to affect these two different dimensions of health has important implications for practice. Furthermore, in our previous analysis of the same data [6], different patterns of association were found between severity of IPV, women's resources, and health, depending on whether mental or physical health was used in the analysis. Thus, to address gaps in the literature previously identified, we tested each study hypothesis twice, using mental health and physical health as dependent variables.

## 3. The Study

### 3.1. Aim and Design

 This paper is a report of an analysis of baseline data from the Women's Health Effects Study (WHES) [6], a prospective, longitudinal investigation of 309 adult, English-speaking Canadian women who, at enrollment, had separated from an abusive partner an average of 20 months previously. See Table 1 for a sample profile. Only 16% of women accessed a shelter in the first 6 months after separating from their abusive partners. Our sample is similar to the general population of Canadian women with respect to educational attainment and percentage reporting aboriginal or racialized status, but women were more economically challenged (i.e., higher rates of unemployment and social assistance, and lower incomes) [46].

After obtaining informed consent, a registered nurse conducted an in-depth structured interview and health assessment to assess women's resources, abuse history, health, service use, and demographic characteristics, supported by computer-assisted data entry (CADE). Interviews were conducted in a private location of the woman's choice in two sessions lasting 60 to 90 minutes each from June 2004–January 2006. Participants were offered a $30 honorarium and reimbursement for childcare and transportation costs.

### 3.2. Ethical Considerations

 A detailed safety protocol guided all interactions between women and the research team. Approval was obtained from the research ethics boards at each site.

### 3.3. Measures

*IPV severity, mental health*, and *physical health* were represented by latent variables, each constructed from three indicators. Factor loading of these indicators on their respective latent constructs were all substantial (0.54. to 0.93) and in the expected direction, providing support for the measurement model. *Social conflict* and *social support* were each manifest variables. The scales used in this study were established, reliable, and valid measures. Internal consistency reliability was acceptable for all scales (i.e., Cronbach's alpha coefficients > 0.80) with the exception of the Gastrointestinal Symptom Subscale of the Partner Abuse Symptom Scale (PASS) [47], a newer scale containing only five items (*α* = 0.65). The use of interviews for data collection resulted in a random pattern of very little missing data. Thus, in computing total scores for each summated rating scale, the woman's mean score for completed items for the scale was substituted for missing values, provided that less than 30% of items had missing values. 

*Severity of IPV*, the intensity of past physical and non-physical violence toward the woman by her expartner, was operationalized using three indicators. The 11-item physical abuse scale of the Index of Spousal Abuse (ISA) [48] captured the severity of physical and sexual battering, and the 19-item nonphysical abuse scale of the ISA was used as an indicator of psychological abuse; the frequency of each abusive act was rated from never (0) to very frequently (4). Using standard scoring, physical and non-physical subscale scores were computed by weighting individual items for severity and summing these scores, for a possible range of 0−100 [48]. The total score from the 10-item Women's Experience of Battering (WEB) scale captured women's experiences of loss of power and control in the context of IPV [49, 50]; items are rated on a six-point Likert scale ranging from strongly agree (1) to strongly disagree (6). 

*Physical health* was operationalized with three indicators to capture both symptom experiences and everyday functioning. First, a general index of past month physical health was created by summing and averaging scores for physical role performance, physical functioning, pain, and general health on the SF12v2 [51]. Second, because chronic pain has been identified as a frequent and often disabling consequence of IPV [52], a measure of pain intensity [53] derived from women's ratings of current pain, worst pain, and average pain in the last 6 months on scales ranging from no pain (0) to the worst pain imaginable (10) was used. Since gastrointestinal disorders are a common symptom reported by women who have experienced IPV, the third indicator was frequency of gastrointestinal symptoms, measured using the five-item gastrointestinal symptom frequency score of the PASS [47]: ratings of the past month frequency of each symptom, using a four-point scale ranging from never (0) to very frequently (4), were summed and averaged for a total score. 

*Mental health* was operationalized with three indicators. The first of these was a general mental health index, created by summing and averaging subscale scores for emotional role performance, vitality, social functioning, and mental health on the SF12v2 [51]. The remaining two indicators were scores on established measures of symptom severity: the 20-item Center for Epidemiologic Studies-Depression Scale (CES-D) [54, 55] and the 17-item Davidson Trauma Scale (DTS), a measure of PTSD symptomology [56]. On the DTS, women who self-identified as having experienced a traumatic event were asked to rate the past week frequency and severity of symptoms consistent with DSM-IV diagnostic criteria for PTSD. Separate frequency and severity scores were computed by summing the responses to applicable items (range 0–56), while an overall score was created by summing the frequency and severity scores (range 0–136). On the CESD, women's rating of the past week frequency of depressive symptoms, on a four-point scale from rarely or none of the time (0) to most of the time (3), was summed to produce total scores (range 0–60). 

*Social support, *the perceived availability of emotional and tangible aid from one's network, and *social conflict, *perceived discord, tension, or stress within these relationships, were measured using separate, 13-item subscales from the InterPersonal Relationship Inventory (IPRI) [30]. The IPRI is based on the assumption that interpersonal relationships within social networks consist of reciprocal exchanges of emotional and tangible resources [29]. Sample social support items include “*There is someone I could go to for anything*” and “*I can talk openly about anything with at least one person I care about*.” Social conflict items include “*Someone I care about takes advantage of me,*” “*There is tension between me and someone I care about,” *and “*People I care about make me do things I do not want to.*” Ratings used a five-point scale, and responses were summed to produce separate scores for social support and social conflict (range 0–65). 

### 3.4. Data Analysis

 Relationships in the hypothesized model (Figure 1) were tested using structural equation modeling (SEM) techniques [57, 58] in AMOS. SEM enables simultaneous testing of direct and indirect or mediating effects in a theoretical model while taking measurement error into account [59, 60]. We tested a series of four models twice, using mental health and physical health as separate dependent variables in each model. First, a single mediator model was tested, specifying a direct relationship between IPV severity and health and an indirect (mediating) effect through either social support (Hypothesis 1) or social conflict (Hypothesis 2), thereby testing the mediating effects of social support and social conflict separately. Next, a double mediator model was tested in which both social support and social conflict were mediators of the relationship between IPV severity and health; this examined whether each variable served as a mediator after controlling for the effects of the other variable (Hypothesis 3). Finally, the moderating effect of social conflict on the relationship between social support and health was tested by dividing the sample into 2 groups (high and low conflict) based on the median score for social conflict and comparing the strength of the path between social support and health across the groups (Hypothesis 4). 

Because a listwise deletion of cases containing missing data would have reduced the sample size by about 16%, models were first estimated using listwise deletion and then reestimated using a full-information, maximum-likelihood (FIML) technique. Given that the parameter estimates were almost identical in magnitude and level of significance, the FIML results are presented here. 

 The fit of the proposed models was assessed using the Comparative Fit Index (CFI) [61], Tucker-Lewis index (TLI) [62, 63], and the Root Mean Square Error of Approximation (RMSEA) [64]. A value of 0.90 or higher for CFI and TLI indicates a good fit between model and data [61–63]. For RMSEA, values under 0.05 indicate very good model fit and 0.05–0.08 indicates reasonably good fit [64]. We show standardized path coefficients (B) to enable comparisons within models and unstandardized coefficients (*β*) to compare the strength of paths between models.

## 4. Results

Table 2 presents descriptive statistics for each of the indicators used in this study. Prior to hypothesis testing, we examined associations between the two primary variables of interest (social support and social conflict) and selected demographic variables. No demographic variables were related to either social support or social conflict with one exception: women with lower incomes reported higher social conflict (*r* = 0.16, *p* = 0.0004), although this relationship was weak.

### 4.1. Single Mediator Models

**Hypothesis**

With physical health as the dependent variable, the social support model was a poor fit with the data (CFI = 0.949, RMSEA = 0.089, TLI = 0.842). *IPV Severity *had a direct negative effect on physical health (*B* = −0.392, *β* = −0.562, *p* < 0.001). Social support had a direct effect on physical health (*B* = −0.229 *β* = −0.540, *p* < 0.001), but no relationship was observed between IPV severity and social support. Thus, social support did not mediate the relationship between IPV severity and physical health. With mental health as the dependent variable, the same model fit the data reasonably well (CFI = 0.985, RMSEA = 0.054, and TLI = 0.966). The direct effect of IPV severity on mental health was −0.296 (*β* = −0.352, *p* < 0.001). Social support had a direct positive effect on mental health (*B* = −0.426, *β* = −0.822, and *p* < 0.001), and this effect was stronger than for physical health. However, no relationship was observed between IPV severity and social support suggesting that social support did not mediate the relationships between IPV severity and mental health. Thus, Hypothesis 1 was not supported.

**Hypothesis 2**

We tested the single mediator models for social conflict using the same approach. Both models fit the data well (CFI = 0.995, RMSEA = 0.033, and TLI = 0.987 for mental health; CFI = 0.973, RMSEA = 0.065, and TLI = 0.937 for physical health). In both models, IPV severity exerted a direct negative impact on health (*B* = −0.366, *β* = −0.535, and *p* < 0.001 for physical health; *B* = −0.262, *β* = −0.311, and *p* < 0.001 for mental health). Significant indirect effects were found for both physical (*B* = −0.039, *β* = −0.058, and *p* < 0.001) and mental health (*B* = −0.064, *β* = −0.076, and *p* < 0.001), but the direct effects were stronger in each model. Social conflict had a direct negative impact on physical (*B* = −0.304, *β* = −0.648, and *p* < 0.001) and mental health (*B* = −0.496, *β* = −0.845, and *p* < 0.001), although the relationships were stronger for mental health. The relationship between IPV severity and social conflict was weak but significant (*B* = −0.130, *β* = −0.090, and *p* < 0.05) in both models. Thus, Hypothesis 2 was supported.

### 4.2. Double Mediator Model

**Hypothesis 3**

**Figure 2:**
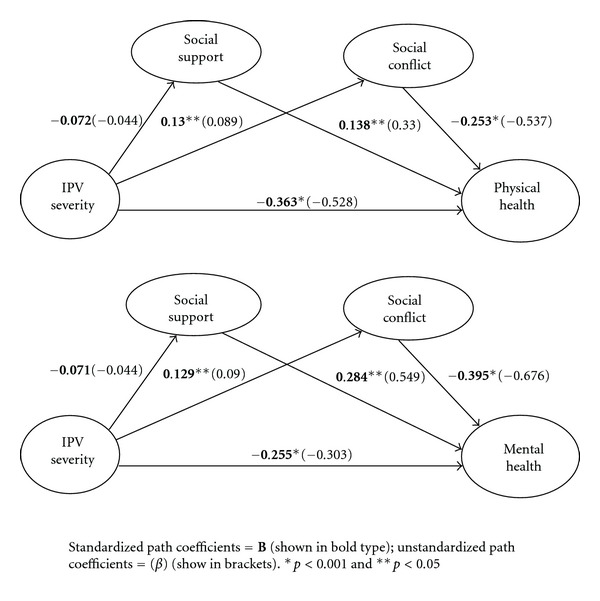
Results of testing the double mediator model (*N* = 309).

Hypothesis 3 was that social support and social conflict simultaneously mediate the relationship between IPV severity and physical and mental health (Figure 2). The mental health model was a good fit (CFI = 0.983,  RMSEA = 0.053, and TLI = 0.963), and the physical health model was an adequate fit (CFI = 0.947, RMSEA = 0.082, and TLI = 0.882). IPV severity exerted a direct significant negative effect on physical (*B* = −0.363, *β* = −0.528, and *p* < 0.001) and mental health (*B* = −0.255, *β* = −0.303, and *p* < 0.001). Social support continued to exert direct positive effects on physical and mental health (*B* = 0.138, *β* = 0.330, and *p* < 0.05; *B* = 0.284, *β* = 0.549, and *p* < 0.001, resp.), while social conflict exerted a negative effect (*B* = −0.253, *β* = −0.537, and *p* < 0.001; and *B* = −0.395, *β* = −0.676, and *p* < 0.001, resp.). No relationship was noted between IPV severity and social support in either the physical health (*B* = −0.072, *β* = −0.044, and *p* < 0.28) or mental health (*B* = −0.071, *β* = −0.044, and *p* < 0.29) model, suggesting a lack of mediation. However, IPV severity had positive effects on social conflict in both the physical and mental health models (*B* = 0.130, *β* = 0.089, and *p* < 0.05; *B* = 0.129, *β* = 0.090, and *p* < 0.05, resp.). Thus, Hypothesis 3 was partially supported: social conflict, but not social support, mediated the relationship between IPV severity and health, after controlling for the effects of social support.

### 4.3. Moderator Model

**Hypothesis 4**

**Figure 3:**
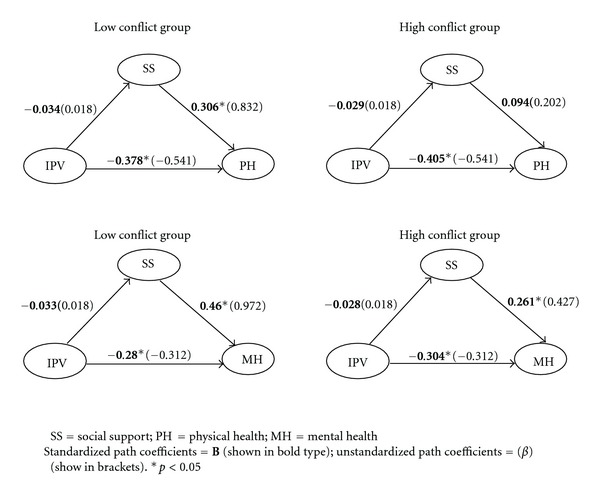
Effects of social support on health for high and low social conflict groups (*N* = 309).

We tested whether social conflict moderated the relationship between social support and health by dividing the sample into low and high social conflict groups and retesting the single mediator models containing social support (Figure 3). The physical health model fit the data well (CFI = 0.971, RMSEA = 0.041, and TLI = 0.945). In the low conflict group, social support was positively associated with physical health (*B* = 0.306, *β* = 0.832, and *p* < 0.001) but this relationship was not significant in the high conflict group (*B* = 0.094, *β* = 0.202, and *p* = 0.286). The magnitude of these paths differed by group (*X* ^2^ = 4.205, *p* = 0.04), suggesting that social conflict moderates the relationship between social support and physical health. Similar results were found for the mental health model which fit the data well (CFI = 0.977, RMSEA = 0.040, and TLI = 0.957). Social support directly affected mental health in the low conflict (*B* = 0.460, *β* = 0.972, and *p* < 0.001) and high conflict groups (*B* = 0.261, *β* = 0.427, and *p* < 0.002) but this effect was stronger in the low conflict group (*X* ^2^ = 5.854, *p* = 0.01). Thus, social conflict attenuated the positive impact of social support on health in both models, supporting Hypothesis 4.

## 5. Discussion

Consistent with the literature, our results reinforce the positive health benefits of social support, but also clarify the interplay between social support and conflict and the potential negative impact of social conflict on women's health. In particular, our results suggest that social conflict affects the health of women who have recently separated from an abusive partner via three pathways: a direct negative effect independent of the abuse experienced; a mediating effect, where more severe IPV generates more social conflict, resulting in poorer health; a moderating effect, whereby the positive impact of social support is reduced in the presence of increased social conflict. These novel results extend the literature and provide balance within research that has overemphasized the positive health impacts of social relationships. This issue is particularly salient for women who are rebuilding their lives after separating from an abusive partner since they experience relationships in both positive and negative ways [15, 16]. 

*The finding that social conflict has a direct, negative impact on women's physical and mental health, regardless of the severity of abuse experienced,* may be explained in terms of stress responses. Women who have separated from an abusive partner experience *conflict* or tension within their social relationships network as *stressful* [15, 16] and chronic stress may lead to various mental and physical health problems [65]. Research has demonstrated that stress and trauma influence cortisol and catecholamine levels, contributing to the persistence of PTSD and even increased cardiovascular disease in trauma survivors [66, 67]. Criticism, conflict, or negative responses to women's experiences of trauma from those in her network have been found to compromise mental health by affecting self-esteem [22, 26]. 

 A novel finding in our study is that social conflict moderated the relationship between social support and health: the positive impact of social support on health was diminished by increased social conflict. This extends Goodman et al. [68] finding that negative reactions from family and friends are related to poorer quality of life after controlling for physical and psychological abuse severity and positive emotional and tangible support. Social support provides clear health benefits to women who have experienced IPV [11, 69, 70]. However, in the presence of increased social conflict, a woman's limited time and energy is diverted to deal with the intrusive costs of social relationships [16]. Thus, women may be less likely to address health problems or make proactive health promotion efforts [44]. 

 Social conflict also acted as a mediator in this study, suggesting that more severe past IPV leads to increased social conflict after leaving, resulting in poorer health. When IPV is more severe, women may have more difficulty disengaging from these relationships because partners are more persistent in their efforts to control and isolate, thereby weakening social ties and increasing tension [23, 71, 72]. When an abusive partner threatens a woman's family and friends, they may avoid the woman due to fear of retribution, increasing old tension or conflicts in these relationships or generating new ones. Goodkind et al. [45] found that women received more negative reactions from family and friends who were threatened by the abuser. Furthermore, family members and friends may lose patience with the women for not leaving sooner. Severe chronic abuse may deplete the woman's social resources due to supporter “burnout” [73, 74]. In this context, women may perceive that friends and family members are critical and nonsupportive [75, 76], leading them to also hide the abuse or minimize its impact. 

 Our finding that social support did not mediate the relationship between IPV severity and health was unexpected, particularly in terms of mental health. The measure of social support (IPRI) used in this study emphasizes emotional support and does not capture economic or instrumental support as well. It is unclear which types of support produce the most health benefits for women while living with an abusive partner and whether this changes after leaving. Given that out participants had left an abusive partner an average of 20 months previously, *past* (compared with more recent, or current) abuse may have exerted less effect on *current* social support than in women who are still living with the abusive partner or had separated more recently. 

### 5.1. Study Limitations

The cross-sectional nature of the data limits the ability to make causal inferences from the results. Further testing of the interrelationships among women's abuse histories, social support, social conflict, and health using longitudinal data will more accurately show the causal mechanisms by which IPV and social resources affect health. Although a diverse community sample of Canadian women took part in this study, further research is also needed to examine the relationships among IPV, women's social resources, and health in more diverse cultural contexts. 

## 6. Implications

 This was the first study to examine the direct, mediating, and moderating effects of social conflict in the context of IPV severity and health using valid measures of social support and social conflict. These findings underscore the importance of considering women's access to social support and its potential health benefits in the context of current social conflict *What are the sources of conflict in women's lives after separation from an abusive partner? How do we as nurses effectively attend to the conflict in the women's lives and what might work in what situations?*

 At a minimum, we suggest that nurses integrate routine assessment of both social support and social conflict into their work with women who have experienced IPV and carefully consider how the expectations they place on women may contribute to their experiences of social conflict. Beyond this, a more radical shift may be needed in both programs and policies from the current emphasis on helping women access services, toward identifying costs of relationships or connections with others (peers and professionals) and developing strategies for managing or dealing with conflict. Further research is needed to develop strategies to help women deal with intrusive social conflict they face and to test the effectiveness of these strategies in improving women's health and quality of life. This type of approach is imbedded within a primary health care intervention for women who have recently left an abusive partner which is currently being tested [77]. 

 All transitions, including disengaging from an abusive partner, bring the potential for increased tension or conflict, as well as positive growth. We know that when leaving abusive partners, women encounter a number of challenges including having to justify separation and/or divorce to their children, family, friends, and others (such as coworkers). Such changes are not always welcomed. Women, who are dealing with high conflict situations in their current social networks in addition to coping with ongoing harassment from previous abusive partners, might not benefit from the usual social support interventions. Health care and social service professionals should pay attention to the types and levels of conflict women are experiencing, who is contributing to such conflict, the most effective ways to deal with such conflict, and the timing of such interventions. The development and evaluation of health interventions which support women's attempts to rebuild new/modified social networks as well as help address the conflicts present or arising out of processes of building such networks are area of future study. 

## Figures and Tables

**Table 1:** Demographic characteristics and abuse histories of study participants (*N* = 309).

Variable	Range	Mean	SD	% of sample
*Demographic characteristics*				
Age	19–63	39.4	9.8	
Education (in years)	6–22	13.4	2.6	
Annual income	0–95,000	15,695	20,391	
Employed				45.1%
Receiving social assistance				31.4%
Disability benefit				10.4%
Living with children (<18 years)				57.0%
Racialized				16.8%
Aboriginal				7.4%
First language english				92.4%
*Abuse History*				
Duration of IPV (in years)	0.25–37	8.5	7.8	
Time since separation (months)	3–40.5	20.1	10.2	
Past month harassment				50%
>1 abusive partner				59%
Abused as a child				81%

**Table 2:** Means, standard deviations and ranges of study indicators (*N* = 309).

Indicator (measure)	Mean	SD	Range
Severity of physical IPV (ISA-P)^1^	48.6	23.47	7.2–100
Severity of nonphysical IPV (ISA-NP)^2^	65.4	18.63	18.5–100
Women's Responses to abuse (WEB)^3^	53.3	7.00	21–60
Social support (IPRI^4^-support scale)	51.6	10.27	16–65
Social conflict (IPRI^4^-conflict scale)	42.0	11.59	13–65
General physical health (SF12v2)	45.3	12.76	14.4–68.7
Chronic pain intensity (Chronic pain scale)	49.0	25.80	0–100
Gastrointestinal symptom frequency (PASS^5^-GI scale)	0.98	0.88	0–4
General mental health (SF12v2)	36.8	12.70	2.7–64.4
Depressive symptom severity (CES-D)^6^	25.2	13.03	0–54.7
PTSD symptomology (DTS)^7^	47.5	30.78	0–125

^1^ISA-P: Index of Spouse Abuse physical scale.

^2^ISA-NP: Index of Spouse Abuse nonphysical abuse scale.

^3^WEB: Women's Experiences of Battering.

^4^IPRI: InterPersonal Relationship Inventory.

^5^PASS: Partner Abuse Symptom Scale, gastrointestinal scale.

^6^CESD: Center for Epidemiologic Studies Depression scale.

^7^DTS: Davidson Trauma Scale.

## References

[1] World Health Organization (2005). *Multi-country Study on Women's Health and Domestic Violence: Report of Initial Results on Prevalence, Health Outcomes and Women's Responses*.

[2] Tjaden P, Thoennes N Extent, Nature and Consequences of Intimate Partner Violence: Findings from the National Violence Against Women Survey.

[3] Golding JM (1999). Intimate partner violence as a risk factor for mental disorders: a meta-analysis. *Journal of Family Violence*.

[4] Campbell JC (2002). Health consequences of intimate partner violence. *The Lancet*.

[5] Ellsberg M, Jansen HA, Heise L, Watts CH, Garcia-Moreno C (2008). Intimate partner violence and women’s physical and mental health in the WHO multi-country study on women’s health and domestic violence: an observational study. *The Lancet*.

[6] Ford-Gilboe M, Wuest J, Varcoe C (2009). Modelling the effects of intimate partner violence and access to resources on women’s health in the early years after leaving an abusive partner. *Social Science and Medicine*.

[7] Kendall-Tackett K (2005). Exciting discoveries on the health effects of family violence: where we are, where we need to go. *Journal of Interpersonal Violence*.

[8] Campbell R, Sullivan CM, Davidson WS (1995). Women who use domestic violence shelters. *Psychology of Women Quarterly*.

[9] Wang JF, McKinney J (1997). Battered women’s perceptions of loss and health. *Holistic Nursing Practice*.

[10] Humphreys J, Lee KA, Neylan TC, Marmar CR (2001). Psychological and physical distress of sheltered battered women. *Health Care for Woman International*.

[11] Coker AL, Watkins KW, Smith PH, Brandt HM (2003). Social support reduces the impact of partner violence on health: application of structural equation models. *Preventive Medicine*.

[12] Belknap J, Melton HC, Denney JT, Fleury-Steiner RE, Sullivan CM (2009). The levels and roles of social and institutional support reported by survivors of intimate partner abuse. *Feminist Criminology*.

[13] Beeble ML, Bybee D, Sullivan CM, Adams AE (2009). Main, mediating, and moderating effects of social support on the well-being of survivors of intimate partner violence across 2 years. *Journal of Consulting and Clinical Psychology*.

[14] Kaslow NJ, Thompson MP, Gibb B (1998). Factors that mediate and moderate the link between partner abuse and suicidal behavior in African American women. *Journal of Consulting and Clinical Psychology*.

[15] Lempert LB (1997). The other side of help: negative effects in the help-seeking processes of abused women. *Qualitative Sociology*.

[16] Wuest J, Ford-Gilboe M, Merritt-Gray M, Berman H (2003). Intrusion: the central problem for family health promotion among children and single mothers after leaving an abusive partner. *Qualitative Health Research*.

[17] Guruge S, Humphreys J (2009). Barriers affecting access to and use of formal social supports among abused immigrant women. *The Canadian Journal of Nursing Research*.

[18] Lindsay AM, Yates BC, Frank-Stronborg M, Olsen SJ (2004). Social support: conceptualization and measurement instruments. *Instruments for Clinical Health-Care Research*.

[19] Berkman LF (1995). The role of social relations in health promotion. *Psychosomatic Medicine*.

[20] Vangelisti AL (2009). Challenges in conceptualizing social support. *Journal of Social and Personal Relationships*.

[21] Underwood PW, Rice VH (2000). Social support: the promise and the reality. *Handbook of Stress, Coping, and Health: Implications for Nursing Research, Theory, and Practice*.

[22] Bradley R, Schwartz AC, Kaslow NJ (2005). Posttraumatic stress disorder symptoms among low-income, African American women with a history of intimate partner violence and suicidal behaviors: self-esteem, social support, and religious coping. *Journal of Traumatic Stress*.

[23] Scarpa A, Haden SC, Hurley J (2006). Community violence victimization and symptoms of posttraumatic stress disorder: the moderating effects of coping and social support. *Journal of Interpersonal Violence*.

[24] Coker AL, Smith PH, Thompson MP, McKeown RE, Bethea L, Davis KE (2002). Social support protects against the negative effects of partner violence on mental health. *Journal of Women’s Health*.

[25] Thompson MP, Kaslow NJ, Kingree JB (2000). Partner violence, social support, and distress among inner-city African American women. *American Journal of Community Psychology*.

[26] Levendosky AA, Bogat GA, Theran SA, Trotter JS, Eye AV, Davidson WS (2004). The social networks of women experiencing domestic violence. *American Journal of Community Psychology*.

[27] Glass N, Perrin N, Campbell JC, Soeken K (2007). The protective role of tangible support on post-traumatic stress disorder symptoms in urban women survivors of violence. *Research in Nursing and Health*.

[28] Carlson BE, McNutt L, Choi DY, Rose IM (2002). Intimate partner abuse and mental health: the role of social support and other protective factors. *Violence Against Women*.

[29] Tilden VP, Nelson CA, May BA (1990). The IPR inventory: development and psychometric characteristics. *Nursing Research*.

[30] Tilden VP, Hirsch AM, Nelson CA (1994). The interpersonal relationship inventory: continued psychometric evaluation. *Journal of Nursing Measurement*.

[31] Tilden VP, Galyen RD (1987). Cost and conflict. The darker side of social support. *Western Journal of Nursing Research*.

[32] Rook KS (1984). The negative side of social interaction: impact on psychological well-being. *Journal of Personality and Social Psychology*.

[33] Pagel MD, Erdly WW, Becker J (1987). Social networks: we get by with (and in Spite of) a little help from our friends. *Journal of Personality and Social Psychology*.

[34] Van Meter MJ, Haynes OM, Kropp JP (1987). The negative social network: when friends are foes. *Child Welfare*.

[35] Harrison MJ, Neufeld A, Kushner K (1995). Women in transition: access and barriers to social support. *Journal of Advanced Nursing*.

[36] Stewart MJ, Tilden VP (1995). The contributions of nursing science to social support. *International Journal of Nursing Studies*.

[37] Davis RC, Brickman E (1996). Supportive and unsupportive aspects of the behavior of others toward victims of sexual and nonsexual assault. *Journal of Interpersonal Violence*.

[38] Rose LE, Campbell J, Kub J (2000). The role of social support and family relationships in women’s responses to battering. *Health Care for Woman International*.

[39] Barnett OW (2001). Why battered women do not leave. Part 2: external inhibiting factors-social support and internal inhibiting factors. Trauma. *Violence and Abuse*.

[40] McLeod A, Hays D, Chang C (2010). Female intimate partner violence survivors’ experiences with accessing resources. *Journal of Counseling and Development*.

[41] Wuest J, Merritt-Gray M (1999). Not going back: sustaining the separation in the process of leaving abusive relationships. *Violence Against Women*.

[42] Riddell T, Ford-Gilboe M, Leipert B (2009). Strategies used by rural women to stop, avoid, or escape from intimate partner violence. *Health Care for Women International*.

[43] Trotter JL, Allen NE (2009). The good, the bad, and the ugly: domestic violence survivors’ experiences with their informal social networks. *American Journal of Community Psychology*.

[44] Ford-Gilboe M, Wuest J, Merritt-Gray M (2005). Strengthening capacity to limit intrusion: theorizing family health promotion in the aftermath of woman abuse. *Qualitative Health Research*.

[45] Goodkind JR, Gillum TL, Bybee DI, Sullivan CM (2003). The impact of family and friends’ reactions on the well-being of women with abusive partners. *Violence Against Women*.

[46] Varcoe C, Hankivsky O, Ford-Gilboe M, Wuest J, Wilk P, Campbell J (2011). Attributing selected costs to intimate partner violence in a sample of women who have left abusive partners: a social determinants of health approach. *Canadian Public Policy*.

[47] Ford-Gilboe M, Campbell J, Merritt-Gray M, Lent B, Samuels-Dennis J, Wilk P Validation of the partner abuse symptom scale (PASS).

[48] Hudson WW, McIntosh SR (1981). The assessment of spouse abuse: two quantifiable dimensions. *Journal of Marriage and the Family*.

[49] Smith PH, Earp JA, DeVellis R (1995). Measuring battering: development of the Women’s Experience with Battering (WEB) Scale. *Women’s Health*.

[50] Smith PH, Smith JB, Earp JAL (1999). Beyond the measurement trap: a reconstructed conceptualization and measurement of woman battering. *Psychology of Women Quarterly*.

[51] Ware JE, Kosinski M, Turner-Bowker DM, Gandek B (2002). *How to Score Version 2 of the SF-12? Health Survey (with a supplement documenting Version 1)*.

[52] Coker AL, Smith PH, Fadden MK (2005). Intimate partner violence and disabilities among women attending family practice clinics. *Journal of Women’s Health*.

[53] Von Korff M, Turk DC, Melzack R (1992). Epidemiological and survey methods: chronic pain assessment. *Handbook of Pain Assessment*.

[54] Comstock GW, Helsing KJ (1976). Symptoms of depression in two communities. *Psychological Medicine*.

[55] Radloff LS (1977). CES-D Scale: a self-report depression scale for research in the general population. *Applied Psychological Measurement*.

[56] Davidson JRT (1996). *Davidson Trauma Scale (DTS)*.

[57] Bollen KA (1989). A new incremental fit index for general structural equation models. *Sociological Methods and Research*.

[58] Kline RB (1998). *Principles and Practice of Structural Equation Modeling*.

[59] Pedhazur E, Smelkin L (1991). *Measurement, Design and Analysis: An Integrated Approach*.

[60] Hoyle R (1995). *Structural Equation modeling: Concepts, Issues and Applications*.

[61] Bentler PM (1990). Comparative fit indexes in structural models. *Psychological Bulletin*.

[62] Tucker LR, Lewis C (1973). A reliability coefficient for maximum likelihood factor analysis. *Psychometrika*.

[63] Bentler PM, Bonett DG (1980). Significance tests and goodness of fit in the analysis of covariance structures. *Psychological Bulletin*.

[64] Browne MW, Cudeck R (1989). Single sample cross-validation indices for covariance structures. *Multivariate Behavioral Research*.

[65] Pearlin L, Aneshensel C, Phelan J (1999). The stress process revisited: reflections on conceptions and their interrelationships. *Handbook of the Sociology of Mental Health*.

[66] Yehuda R (2001). Biology of posttraumatic stress disorder. *Journal of Clinical Psychiatry*.

[67] Violanti JM, Hartley TA, Charles LE (2006). Police trauma and cardiovascular disease: association between PTSD symptoms and metabolic syndrome. *International Journal of Emergency Mental Health*.

[68] Goodman E, Huang B, Wade TJ, Kahn RS (2003). A multilevel analysis of the relation of socioeconomic status to adolescent depressive symptoms: does school context matter?. *Journal of Pediatrics*.

[69] Meadows LA, Kaslow NJ, Thompson MP, Jurkovic GJ (2005). Protective factors against suicide attempt risk among African American women experiencing intimate partner violence. *American Journal of Community Psychology*.

[70] Theran SA, Sullivan CM, Bogat GA, Stewart CS (2006). Abusive partners and ex-prtners: understanding the effects of relationship to the abuser on women’s well-being. *Violence Against Women*.

[71] Kocot T, Goodman L (2003). The roles of coping and social support in battered women’s mental health. *Violence Against Women*.

[72] Fowler DN, Hill HM (2004). Social support and spirituality as culturally relevant factors in coping among African American women survivors of partner abuse. *Violence Against Women*.

[73] Lepore SJ, Evans GW, Schneider ML (1991). Dynamic role of social support in the link between chronic stress and psychological distress. *Journal of Personality and Social Psychology*.

[74] Kaniasty K, Norris FH (1993). A test of the social support deterioration model in the context of natural disaster. *Journal of Personality and Social Psychology*.

[75] Herman JL (1997). *Trauma and Recovery: The Aftermath of Violence—From Domestic Abuse to Political Terror*.

[76] El-Bassel N, Gilbert L, Rajah V, Foleno A, Frye V (2001). Social support among women in methadone treatment who experience partner violence: isolation and male controlling behavior. *Violence Against Women*.

[77] Ford-Gilboe M, Merritt-Gray M, Varcoe C, Wuest J (2011). A theory-based primary health care intervention for women who have left abusive partners. *Advances in Nursing Science*.

